# Supplementary material for: Complex fault interaction controls continental rifting
Source: Nat Commun. 2017 Oct 30;8:1179. doi: 10.1038/s41467-017-00904-x (PMC5661748; doi:10.1038/s41467-017-00904-x)
Supplement: Supplementary file 1 — Supplementary Information [file 41467_2017_904_MOESM1_ESM.pdf]

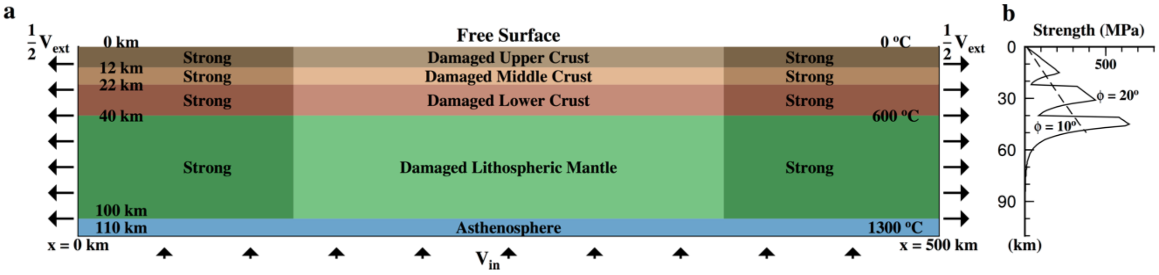


**Supplementary Figure 1**.

**Numerical Setup**. **a**, Model geometry, internal composition, boundary and initial conditions. **b,** Initial strength profile assuming a uniform strain rate of $10^{-14}$ $s^{-1}$ and an internal friction angle of 20$^{o}$ or 10$^{o}$. See Methods for additional details.

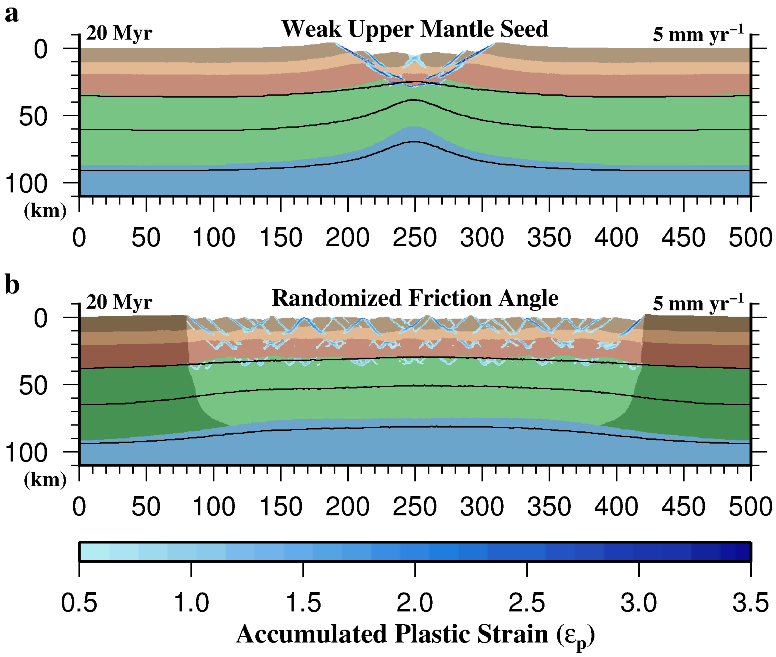


**Supplementary Figure 2**

**Effect of Initial Seed Type.** Deformation patterns after 20 Myr of extension (5 mm $yr^{-1}$) using a week seed (**a**) or randomly distributed heterogeneities (**b**). The model spatial resolution is 500 m. The weak seed (**a**) is placed 2 km beneath the Moho in the model center, has dimensions of 4x6 km and contains the same properties as the surrounding mantle except for the initial internal friction angle and cohesion, which are set to weakened values (10$^{o}$, 20 MPa).

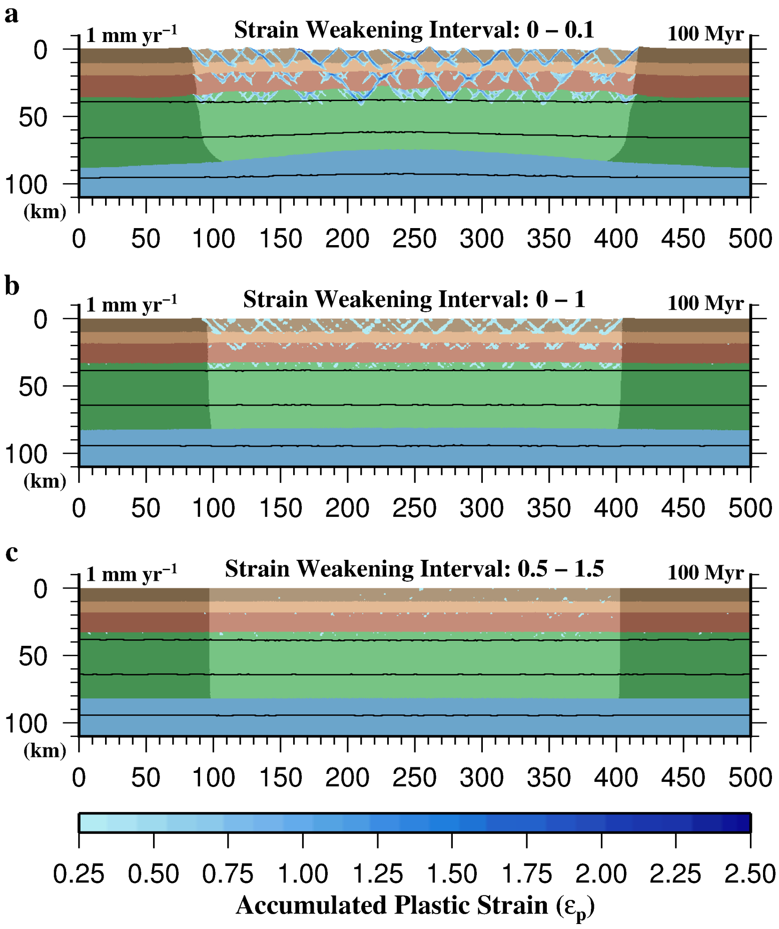


**Supplementary Figure 3**

**Effect of Strain-Weakening Rate.** Effect of the rate of strain-weakening on deformation patterns at a velocity of 1 mm $yr^{-1}$ (stretching phase) after 100 Myr of extension. Deformation initiates on randomly distributed heterogeneities. At rapid rates of strain-weakening rates (0-0.1, **a**), deformation quickly localizes onto high-angle faults in brittle portions of the lithosphere and significant amounts of plastic strain accumulate. As the rate of strain-weakening is slowed to between **(b)** 0-1 and (**c**) 0.5-1.5, minimal to almost no brittle deformation accumulates along discrete faults and deformation is accommodated by lithospheric stretching. The model spatial resolution is 500 m. The models presented in the main text (Fig. 2-5) use strain-weakening rates of 0-0.1, but have a higher spatial resolution (250 m). Black lines mark temperature contours of 600, 900 and 1200 $^{o}$C.

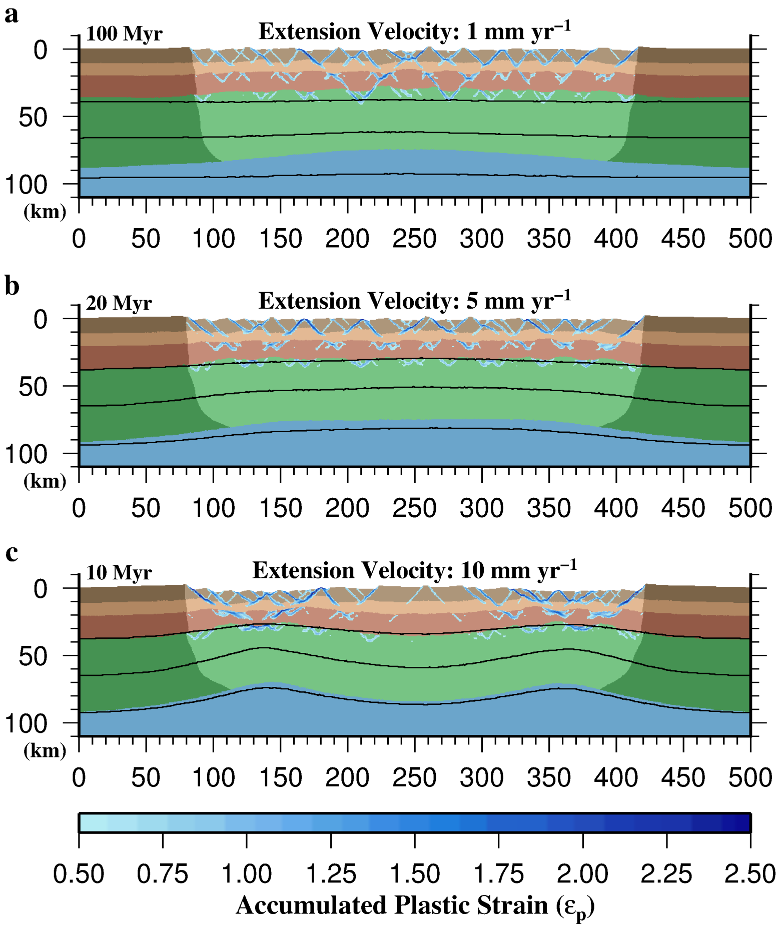


**Supplementary Figure 4**

**Influence of initial stretching phase velocity.** Deformation patterns for an initial velocity of 1, 5 or 10 mm $yr^{-1}$. Deformation initiates on randomly distributed heterogeneities (0-0.1 strain weakening interval) and the model spatial resolution is 500 m. Black lines mark temperature contours of 600, 900 and 1200 $^{o}$C. At extension velocities up to 5 mm $yr^{-1}$ (**a-b**), deformation is able to localize onto distributed discrete faults within brittle layers of the lithosphere, which accommodate uniform thinning of the crust for extended periods (20-100 Myr). At higher extension velocities (**c**) deformation still localizes onto discrete faults, but lithospheric necking also rapidly initiates within a few Myr, which is inconsistent with observations of the initial stretching phase of continental breakup. The model stage representing the stretching phase presented in the main text (Fig. 2a) has an extension velocity of 1 mm $yr^{-1}$.

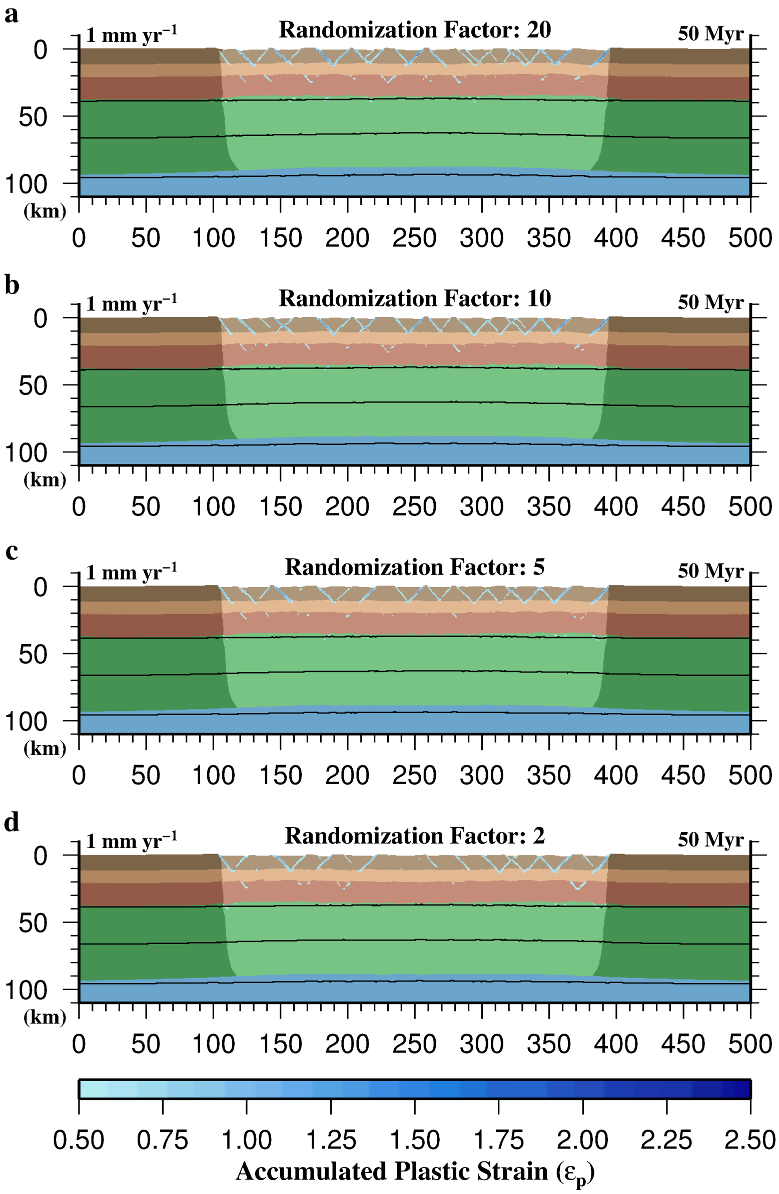


**Supplementary Figure 5**

**Effect of mechanical randomization magnitude on the stretching deformation phase**. Deformation patterns at the end (50 Myr) of the stretching phase (1 mm $yr^{-1}$) for various values of the internal friction angle randomization factor *F* (see Methods). The model spatial resolution is 500 m. In comparison to the value of *F* (10, panel **b**) used in Fig. 2-5 and Supplementary Fig. 2-4,7-13, increasing the randomization factor to 20 (**a**) slightly increases the number of faults in the uppermost brittle crust. Similarly, decreasing *F* to 5 (**c**) or 2 (**d**) produces slightly fewer upper crustal faults. Aside from this change in the number of faults, the model simulations are largely identical.

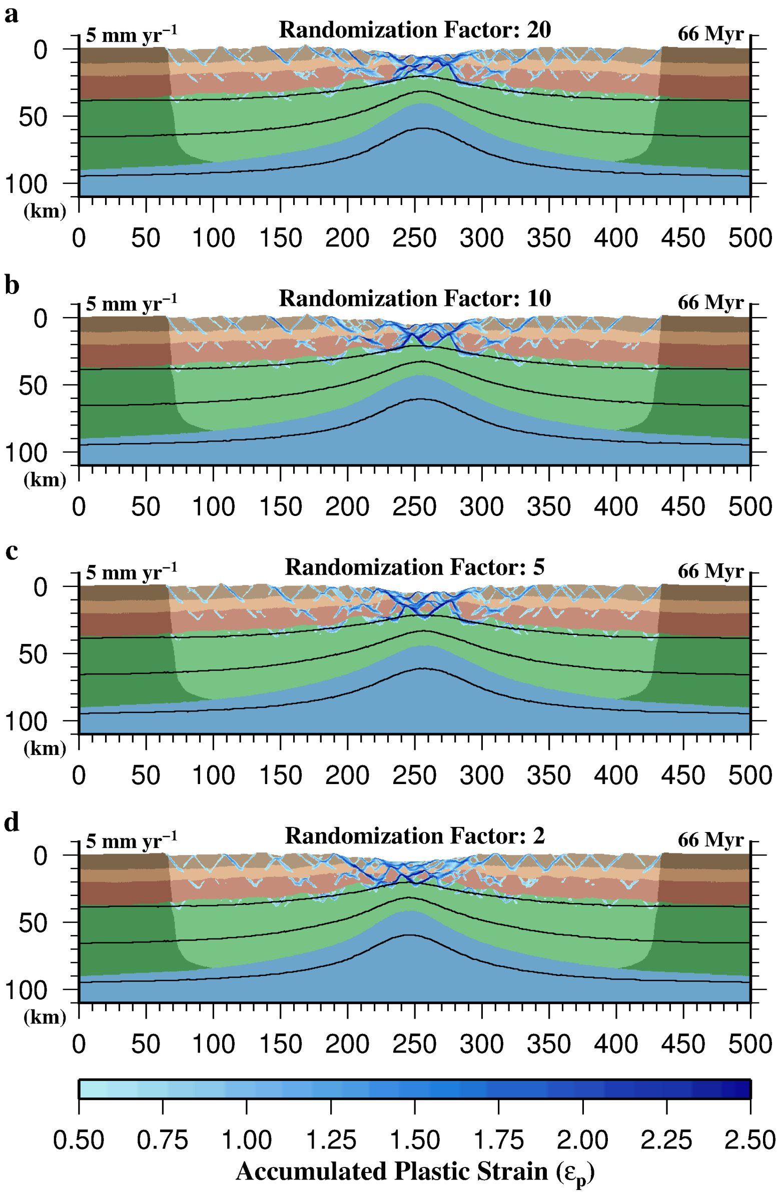


**Supplementary Figure 6**

**Effect of mechanical randomization magnitude on the thinning and hyperextension deformation phase.** Deformation patterns during the hyperextension phase (5 mm $yr^{-1}$) for various values of the internal friction angle randomization factor *F* (see Methods). While a decrease in the randomization factor from 20 to 2 produced a slight decrease in the number of stretching phase faults (Supplementary Figure 5), the deformation patterns after significant lithospheric thinning show similar structures and patterns of complexity in the necking and distal domains.

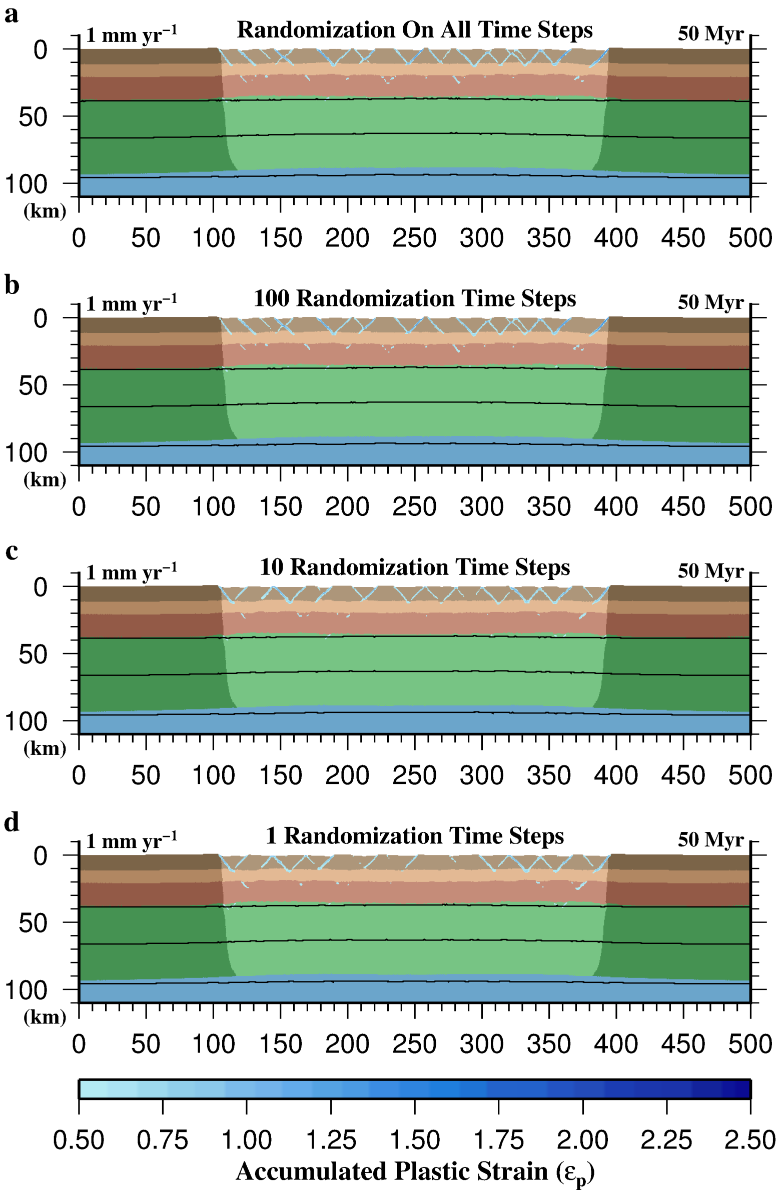


**Supplementary Figure 7**

**Effect of mechanical randomization duration on the stretching deformation phase**. Deformation patterns at the end (50 Myr) of the stretching phase (1 mm yr$^{-1}$) for an internal friction angle randomization factor (*F*) of 10 that is applied for all (**a**), 100 (**b**), 10 (**c**) or 1 (**d**) time steps. As the time step is fixed at 100,000 years until the model reaches 50 Myr (after that the model time step is 20,000 years), 100 time steps correspond to 10 Myr. Limiting the number randomization time steps to 100 (**b**) produces very similar results to the simulation with randomization at every time step (**a**). Further decreasing the number of randomization steps produces slightly fewer stretching phase faults and comparatively less accumulated plastic strain along each fault strand. As in previous supplementary figures, the model spatial resolution is 500 m.

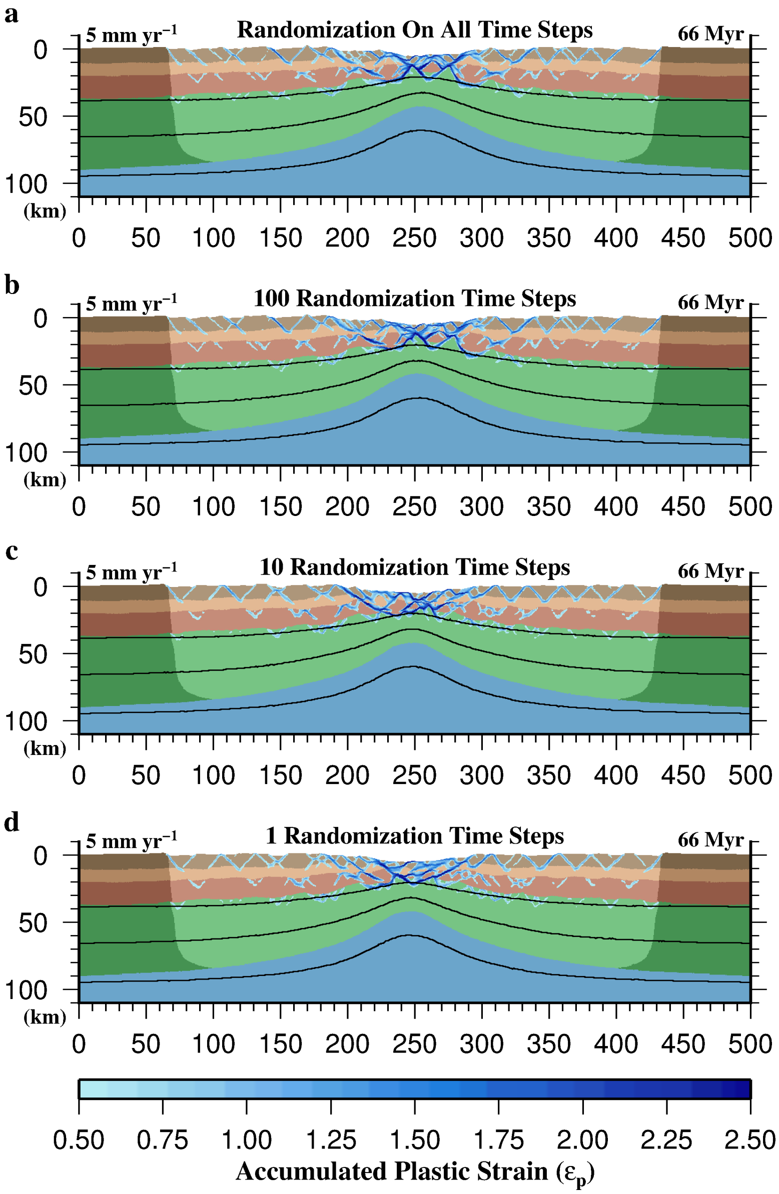


**Supplementary Figure 8**

**Effect of mechanical randomization duration on the thinning and hyperextension deformation phase.** Deformation patterns during the hyperextension phase (5 mm yr$^{-1}$) for an internal friction angle randomization factor (*F*) of 10 that is applied for all (**a**), 100 (**b**), 10 (**c**) or 1 (**d**) time steps (see Supplementary Figure 7). Except for panel **a**, randomization ended during the stretching phase for all other models. As observed in Supplementary Figure 6, mechanical randomization for all or 100 time steps produces very similar results in terms of both broad structural geometries and specific deformation patterns within the necking zone. Randomization for 10 or 1 time steps also produces broadly similar first-order features, but the extent of crustal thinning is reduced as the randomization time step decreases. Effectively, fewer faults with less accumulated strain in the stretching phase leads to a slight delay in the progression of thinning, hyperextension-exhumation and breakup.

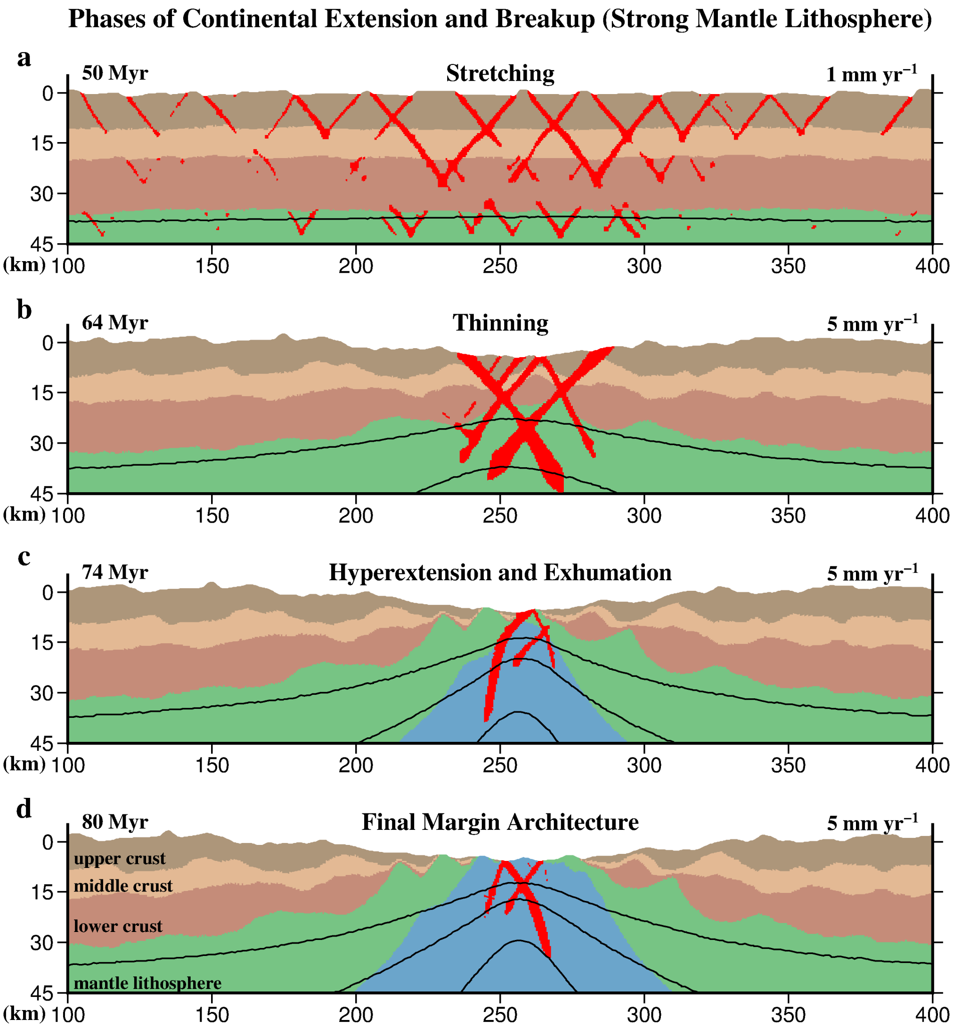


## Supplementary Figure 9

**Evolution of rifting phases at a reduced spatial resolution.** Results for a model identical to the one presented in Figures 2-5, except the spatial resolution is reduced from 250 m to 500 m grid spacing. The panels correspond directly to Figure 1**e-h**. 'Strong mantle lithosphere' refers to the use of a dry olivine flow law, which contrasts with Supplementary Figures 11-12 where the mantle lithosphere follows a wet olivine flow law.

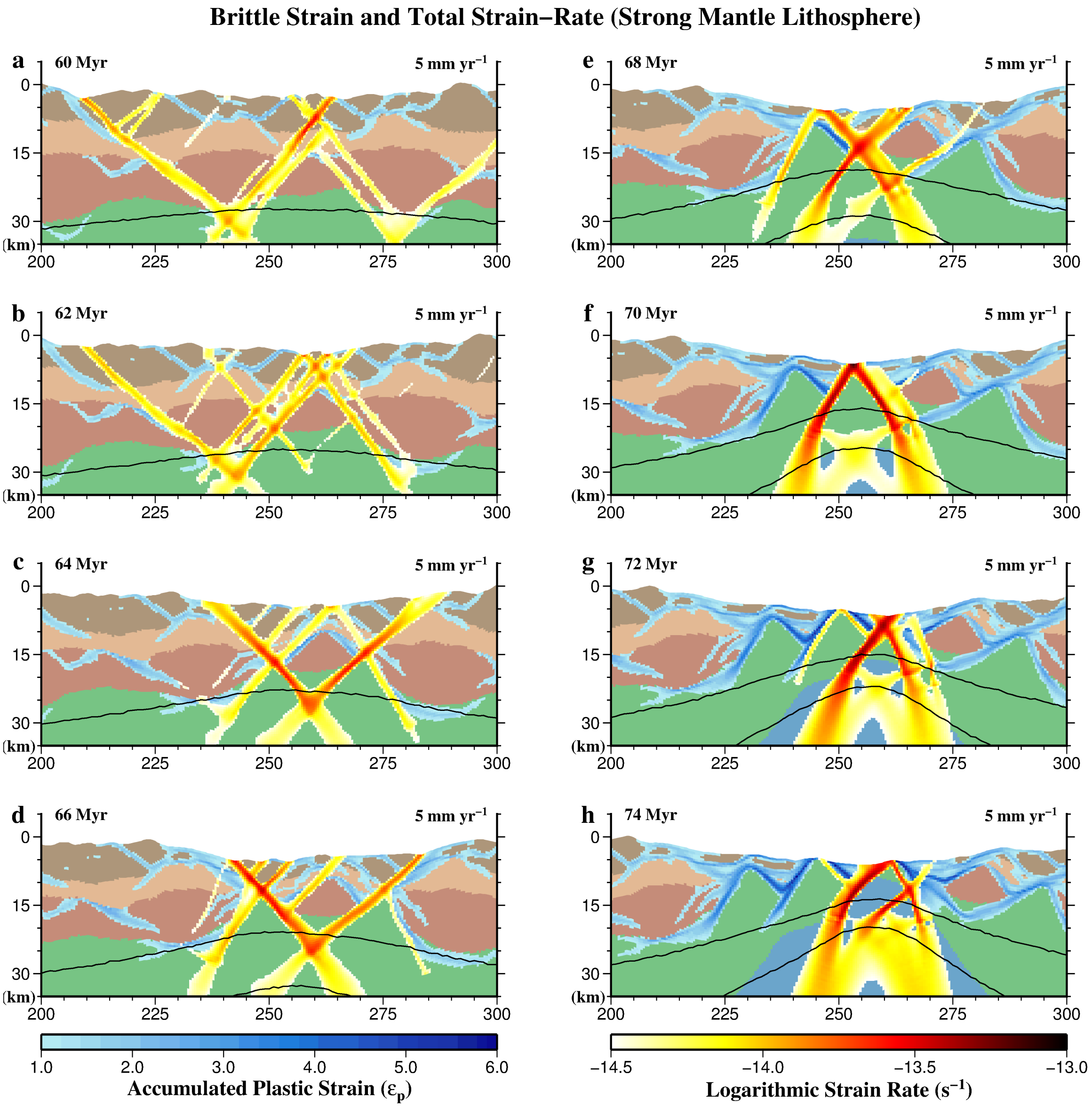


## Supplementary Figure 10

**Evolution of accumulated and active deformation at a reduced spatial resolution.** Results for a model identical to the one presented in Fig. 1-4, except the spatial resolution is reduced from 250 m to 500 m grid spacing. The results correspond directly to Figure 3 (250 m spatial resolution model). As in supplementary Figure 9, ‘Strong mantle lithosphere’ refers to the use of a dry olivine flow law.

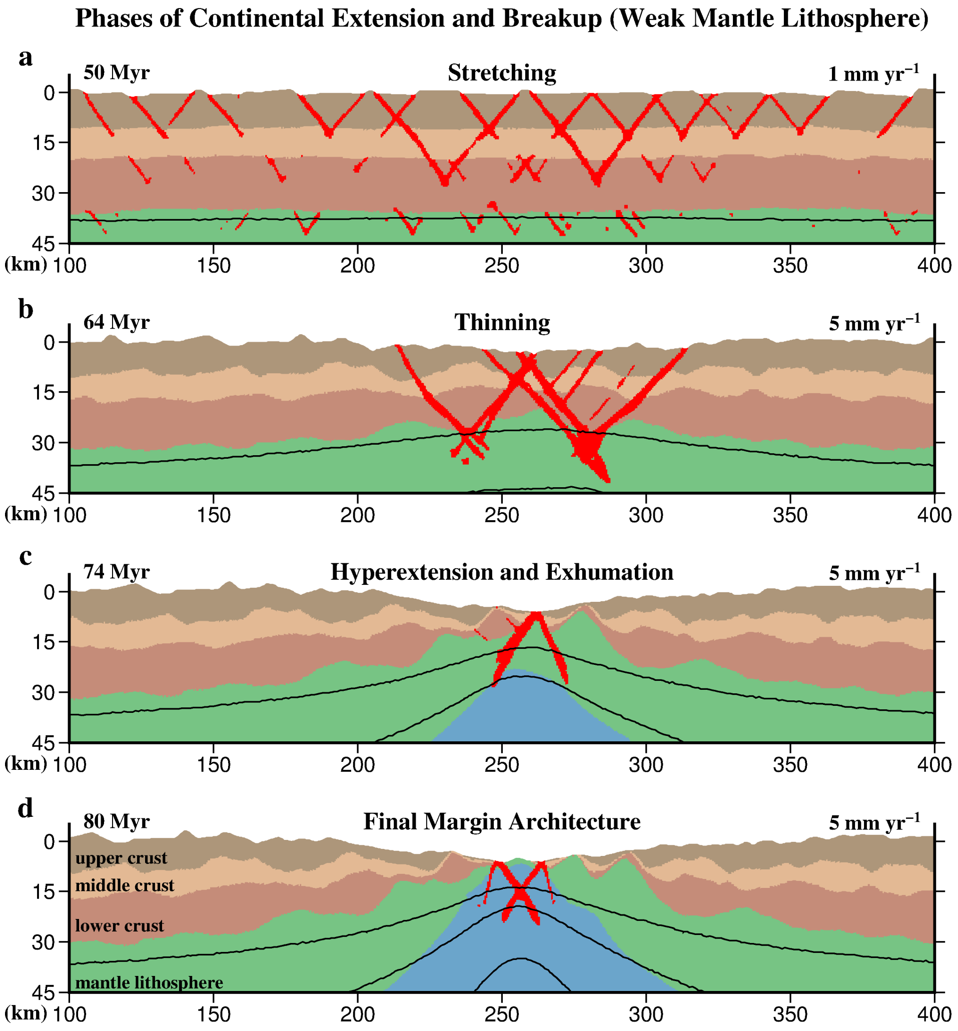


**Supplementary Figure 11**

**Evolution of rifting phases with a weaker mantle lithosphere.** Results for a model identical to the one in Supplementary Figures 9-10, except a mechanically weaker (i.e., lower viscosity) wet olivine flow law[36] is used in place of a dry olivine flow law for the mantle lithosphere. The primary effect of weakening the mantle lithosphere is to delay mantle necking and eventual breakup of the lithosphere.

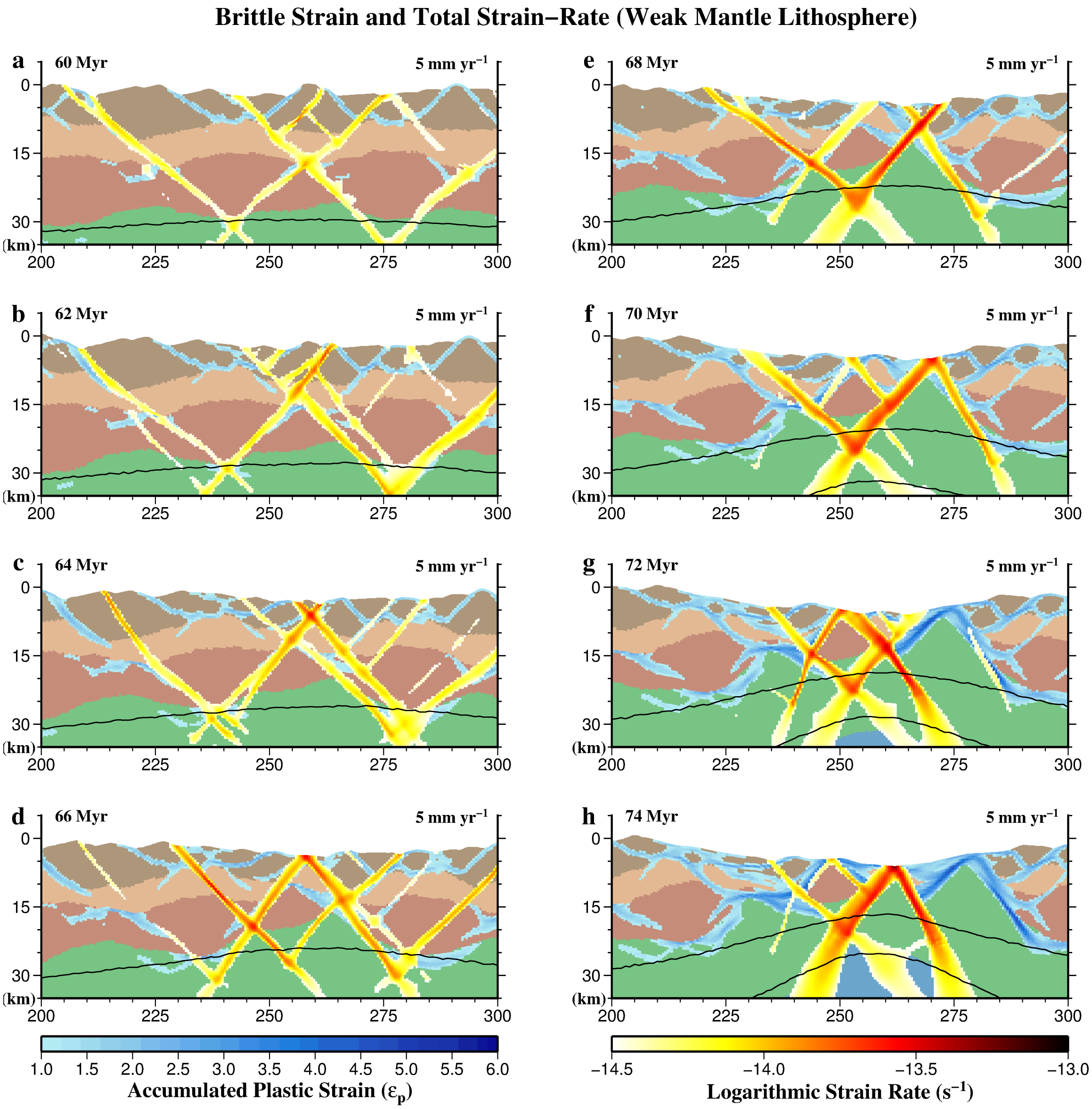


## Supplementary Figure 12

**Evolution of accumulated and active deformation with a weaker mantle lithosphere.** Detailed evolution of the thinning and hyper-exhumation phases for a model with a lithospheric mantle governed by a weaker (wet olivine) mantle lithosphere.

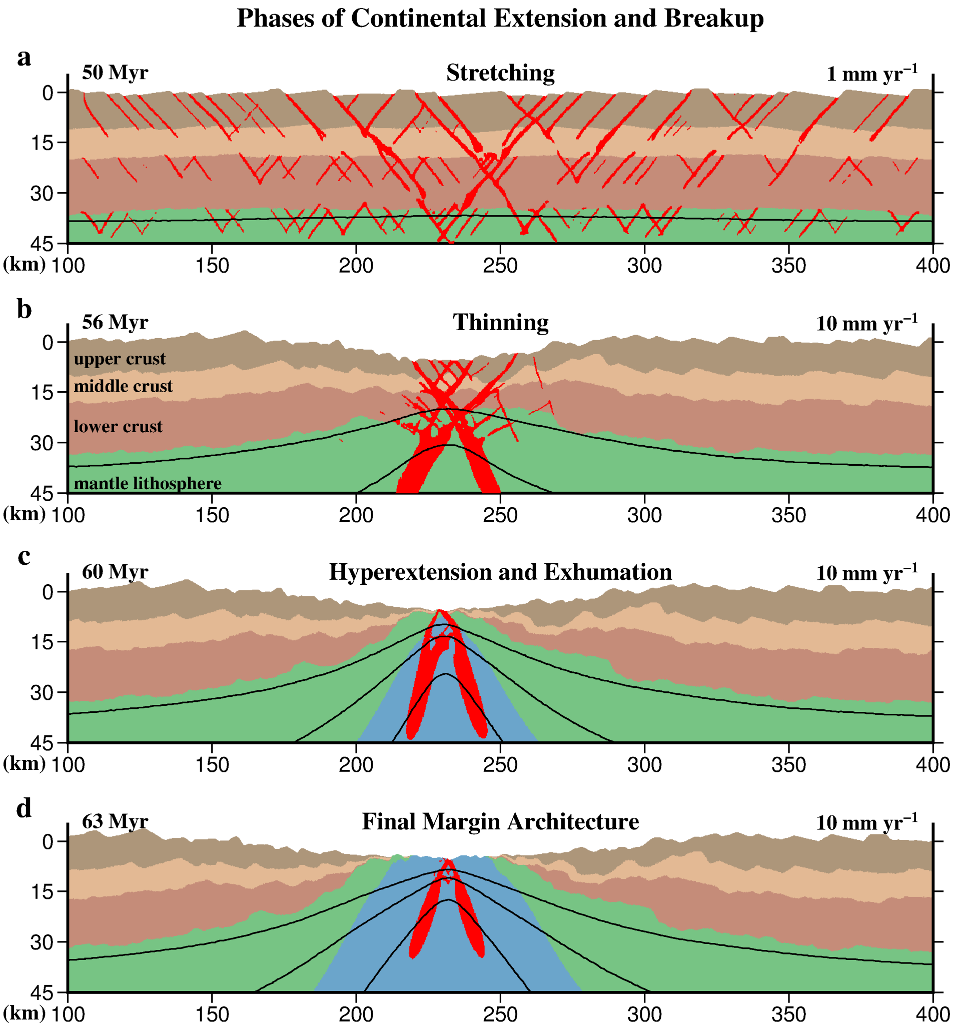


**Supplementary Figure 13**

**Evolution of rifting with a fast phase velocity of 10 mm yr$^{-1}$.** Phases of continental extension and rifted margin formation that correspond the conceptual model shown in Fig. 1. While the deformation field at 50 Myr (**a**, end of stretching phase) is identical to that in Fig. 2a, the velocity from 50 Myr on is 10 mm yr$^{-1}$ in comparison to 5 mm yr$^{-1}$ in Fig. 2-5. This relative increase in velocity predictably leads to more rapid continental rifting, with the time between each descriptive phase (**b**, Thinning, **c**, Hyperextension-Exhumation) significantly reduced. The width of the active deformation zone in the thinning phase is also somewhat narrower. The model spatial resolution in this simulation is 250 m.

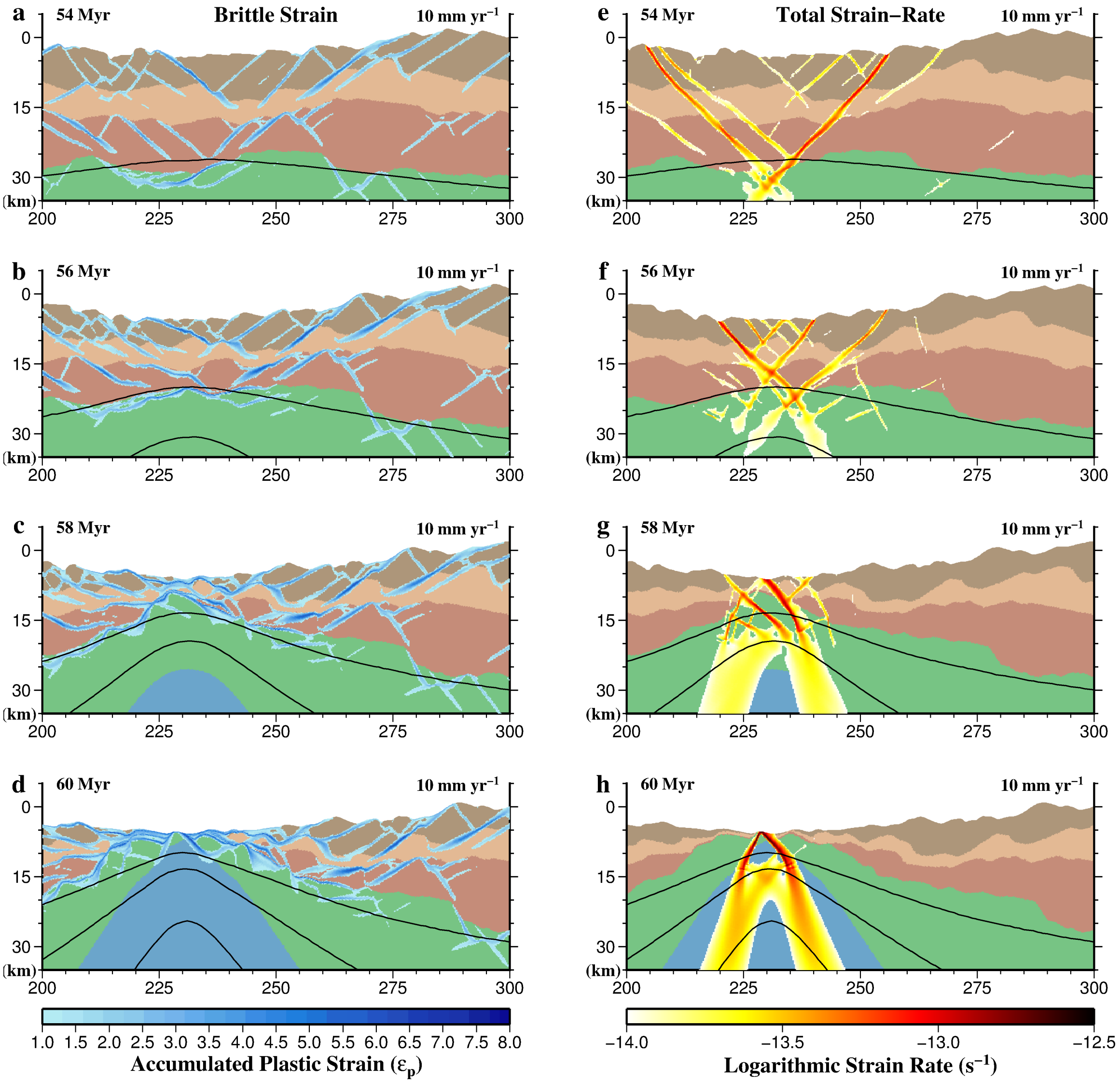


## Supplementary Figure 14

**Evolution of accumulated and active deformation with a fast phase velocity of 10 mm yr$^{-1}$**. Deformation fields from 54-60 Myr (2 Myr increments) highlighting the transition from thinning to exhumation for a velocity of 10 mm yr$^{-1}$. **a-d**, Accumulated plastic strain illustrates the evolution of both active and deactivated fault structures. **e-h**, Logarithmic strain rate illustrates the location of active deformation. Fig. 3 in the main text illustrates the equivalent fields for a fast phase velocity of 5 mm yr$^{-1}$. In comparison to models with a fast phase velocity of 5 mm yr$^{-1}$ (Fig. 2-5), deformation localizes quickly into a narrow rifting region (panels **b-c**, **f-g**) and exhumation of the mantle lithosphere (panels **d**, **h**) occurs roughly 10-12 Myr earlier.

## Supplementary Table 1

| Parameter | Units | Upper Cont. Crust | Mid. Cont. Crust | Lower Cont. Crust | Lithospheric Mantle | Sub- Lithospheric Mantle |
|---|---|---|---|---|---|---|
| Density ($\rho_o$) | kg $m^{-3}$ | 2800 | 2850 | 2900 | 3250 | 3300 |
| Flow Law[a] | - | Wet Qtz. | Wet Qtz. | Wet Anth. | Dry Olivine (*disl*) | Dry Olivine (*diff,disl*) |
| Visc. Prefactor ($A^*$)[b] | $Pa^{-n}$ $m^{-p}$ $s^{-1}$ | $8.57 \times 10^{-28}$ | $8.57 \times 10^{-28}$ | $7.13 \times 10^{-18}$ | $6.52 \times 10^{-16}$ | $2.37 \times 10^{-15}$ , $6.52 \times 10^{-16}$ |
| n | - | 4 | 4 | 3 | 3.5 | 1, 3.5 |
| Activation energy ($Q$) | kJ $mol^{-1}$ | 223 | 223 | 345 | 530 | 375, 530 |
| Activation volume ($V$) | $m^3$ $mol^{-1}$ | - | - | - | $18 \times 10^{-6}$ | $10 \times 10^{-6}$, $18 \times 10^{-6}$ |
| Specific heat ($C_p$) | J $kg^{-1}$ $K^{-1}$ | 750 | 750 | 750 | 750 | 750 |
| Thermal conductivity (k) | W $m^{-1}$ $K^{-1}$ | 2.5 | 2.5 | 2.5 | 2.25 | 39.25 |
| Thermal expansivity ($\alpha$) | $K^{-1}$ | 0 | 0 | 0 | $2.5 \times 10^{-5}$ | $2.5 \times 10^{-5}$ |
| $T_0$ | $^oC$ | - | - | - | 600 | 600 |
| Heat production (H) | W $m^{-3}$ | $9 \times 10^{-7}$ | $9 \times 10^{-7}$ | $9 \times 10^{-7}$ | 0 | 0 |
| Grain Size (d) | m | - | - | - | - | 5.e-3 , - |
| Grain Size Exponent (p) | - | - | - | - | - | 3 , - |

**Material properties.** Viscous flow law properties, material densities and thermodynamic properties for different model compositions (Supplementary Fig. 1). Viscous flows contained within the model include wet quartzite[34] (upper- and middle- continental crust), wet anorthite[35] and dry olivine[36] (lithospheric mantle and sub-lithospheric mantle).
