# Supplementary material for: Complex fault interaction controls continental rifting
Source: Nat Commun. 2017 Oct 30;8:1179. doi: 10.1038/s41467-017-00904-x (PMC5661748; doi:10.1038/s41467-017-00904-x)
Supplement: Supplementary file 3 — Description of Additional Supplementary Files [file 41467_2017_904_MOESM3_ESM.pdf]

Description of Additional Supplementary Files

File Name: Supplementary Movie 1
Description: **Detailed evolution of accumulated and active deformation during the thinning and hyperextension-exhumation phases.** Animation showing the evolution of the model in Fig. 2-5 at increments of 100,000 years. As in Fig. 4 and 5, each frame shows the logarithmic strain-rate and plastic strain on top of the background compositional field.
